# Nutritional Needs and Support for Children with Chronic Liver Disease

**DOI:** 10.3390/nu9101127

**Published:** 2017-10-16

**Authors:** Christine H. Yang, Brandon J. Perumpail, Eric R. Yoo, Aijaz Ahmed, John A. Kerner

**Affiliations:** 1 Division of Pediatric Gastroenterology, Hepatology and Nutrition, Lucile Packard Children’s Hospital, Palo Alto, Stanford, CA 94304, USA; christine.yang@stanford.edu (C.H.Y.); JKerner@stanfordhealthcare.org (J.A.K.J.); 2 Department of Medicine, Drexel University College of Medicine, Philadelphia, PA 19129, USA; bjp63@drexel.edu; 3 Department of Medicine, Santa Clara Valley Medical Center, San Jose, CA 95128, USA; eric.r.yoo@gmail.com; 4 Division of Gastroenterology and Hepatology, Stanford University School of Medicine, Stanford, CA 94305, USA

**Keywords:** nutrition, chronic liver disease, children

## Abstract

Malnutrition has become a dangerously common problem in children with chronic liver disease, negatively impacting neurocognitive development and growth. Furthermore, many children with chronic liver disease will eventually require liver transplantation. Thus, this association between malnourishment and chronic liver disease in children becomes increasingly alarming as malnutrition is a predictor of poorer outcomes in liver transplantation and is often associated with increased morbidity and mortality. Malnutrition requires aggressive and appropriate management to correct nutritional deficiencies. A comprehensive review of the literature has found that infants with chronic liver disease (CLD) are particularly susceptible to malnutrition given their low reserves. Children with CLD would benefit from early intervention by a multi-disciplinary team, to try to achieve nutritional rehabilitation as well as to optimize outcomes for liver transplant. This review explains the multifactorial nature of malnutrition in children with chronic liver disease, defines the nutritional needs of these children, and discusses ways to optimize their nutritional.

## 1. Introduction

The liver plays a crucial role in many of the body’s metabolic processes, including regulating protein, fat, and carbohydrate metabolism; vitamin storage and activation; and detoxification and excretion of waste products [1]. In children with chronic liver disease (CLD), disruption of these processes results in improper nutrient digestion, absorption, and usage, and ultimately malnutrition. CLD is defined as progressive destruction and regeneration of liver parenchyma leading to fibrosis and cirrhosis, which has been present for at least six months [2]. It results from hepatic injury leading to irreversible impairment of liver function, with changes in architecture and blood supply [3]. This results in impaired synthesis of serum proteins and clotting factors, compromised glycemic control and ammonia metabolism, and impaired bile secretion and cholestasis [4]. The prevalence and etiologies for CLD in children can vary by country and age of onset. In the United States, the prevalence of liver disease in children is unclear; however, it is estimated that 15,000 children are hospitalized for liver disease annually [5]. The overall incidence of liver disease in neonates in the United States is approximately 1 in every 2500 live births [6] with extrahepatic biliary atresia, metabolic disorders, and neonatal hepatitis being the most common causes of CLD in neonates [7]. In older children in the United States, common causes of CLD include metabolic disorders, chronic intrahepatic cholestasis, obesity-related steatohepatitis, drug- and toxin-induced disorders, and viral hepatitis [5]. In Australia, the most common cause of CLD starting in the neonatal age in children is biliary atresia, occurring in approximately 1 in 8000 live births, with other common causes being alpha-1-antitrypsin deficiency and Alagille’s syndrome [8,9]. Similarly, in Brazil, biliary atresia is the most common cause of CLD in children [10]. In contrast, a study in Pakistan found viral hepatitis to be the most common cause of neonatal onset CLD, followed by metabolic disorders and biliary atresia [11] while a study in India found metabolic disorders to be the most common cause of CLD in children [12].

Children are particularly susceptible to malnutrition due to their high energy needs for growth [13]. Approximately 25% of children diagnosed with CLD worldwide are undernourished, with the incidence being higher in developing countries [14]. Furthermore, many children with chronic liver disease will eventually require liver transplantation, and studies have noted that malnourishment during liver transplantation is associated with poorer outcomes, including increased risks for morbidity and mortality [15,16,17] as well as compromised neurocognitive development [18,19] and growth [20]. Otherwise unexplained clinical or laboratory nutritional deficiencies (such as iron deficiency, vitamin D deficiency/rickets, vitamin K deficiency/coagulopathy) should raise suspicion for CLD potentially complicating another diagnosis, such as celiac disease, inflammatory bowel disease, or chronic cholestasis [21]. This review discusses the multi-factorial mechanisms behind malnutrition in children with CLD, as well as strategies to optimize nutritional support in children with CLD.

## 2. Mechanisms of Malnutrition in Children with Cld

### 2.1. Decreased Energy Intake

**Figure 1:**
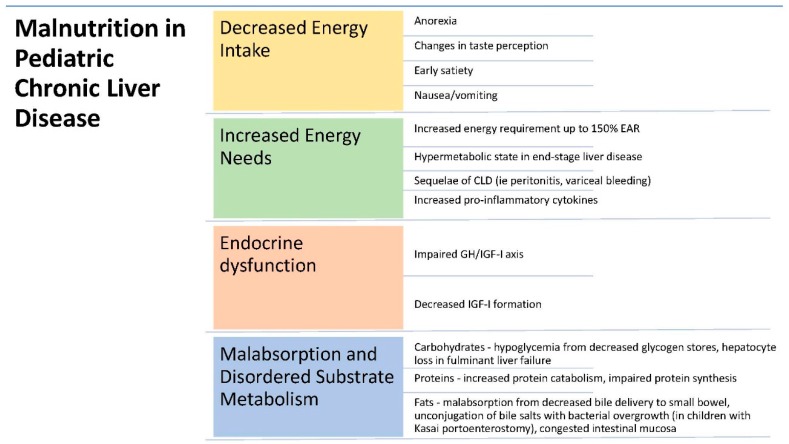
Mechanisms of malnutrition in children with CLD.

Children with CLD are often unable to consume adequate calories for their energy needs (Figure 1) [22]. Contributing factors include anorexia, changes in taste perception, early satiety, and nausea and vomiting [13]. Anorexia is attributed to changes in amino acid metabolism, which results in increased tryptophan levels and subsequent increases in brain serotonergic activity. Tryptophan is the amino acid precursor to serotonin, which regulates eating behavior. Increased cerebrospinal concentrations of tryptophan have been associated with anorexia in patients with CLD [23]. Deficiency in zinc or magnesium contributes to changes in taste perception, which may be aggravated by supplementation with unpalatable formulas and also discourages intake [13]. Furthermore, pediatric CLD patients have decreased stomach volume and discomfort from ascites and organomegaly that result in early satiety [24]. Increased pro-inflammatory cytokines, common in children with CLD, results in nausea and vomiting [25]. Together, these factors lead to decreased consumption and reduced energy intake.

EAR: estimated average requirements; GH: growth hormone; IGF: insulin-like growth factor.

### 2.2. Increased Energy Needs

Children with CLD can have an increase in energy requirements of up to 140% compared to children without CLD [26,27,28]. Children with end-stage liver disease in particular are in a hypermetabolic state, in which there is increased metabolic activity and excess lipid oxidation [29]. Moreover, this is further aggravated by the sequelae of CLD, including episodes of sepsis from peritonitis or cholangitis, as well as variceal bleeding [13,30]. Children with CLD can also have higher levels of pro-inflammatory cytokines, which have been correlated with malnutrition due to increased energy consumption [31]. 

### 2.3. Endocrine Dysfunction

In addition to decreased energy intake and increased metabolism, growth failure in children with CLD is further aggravated by an impaired growth hormone (GH)/insulin-like growth factor (IGF-I) axis. IGF-I and its major circulating binding protein, IGF Binding Protein 3 (IGF-BP3), are synthesized in the liver, and protein malnutrition decreases IGF-I formation, as well as increases its serum clearance and degradation [32]. IGF-I levels are decreased further due to GH resistance in children with CLD caused by downregulation of the GH receptor [30].

### 2.4. Malabsorption and Disordered Substrate Metabolism

#### 2.4.1. Carbohydrates

The liver receives glucose-rich blood via the portal vein, from which it creates glycogen to be stored in the liver. Glucose is also circulated from the liver to the muscles where lactate, pyruvate, and alanine are generated via glycolysis [33,34]. However, in children with CLD, glycogen stores are depleted from their longstanding condition, resulting in hypoglycemia. Significant hepatocyte loss, such as that in fulminant liver failure, can also cause hypoglycemia [35]. Infants and small children are particularly susceptible due to their lower reserve [13]. In contrast, adults with cirrhosis have increased insulin levels and can develop diabetes mellitus due to increased insulin secretion by the pancreas, decreased insulin degradation by the liver [36] and decreased glucose uptake by tissues [37]. These processes may also occur in children, but unlike adults, few children with CLD (aside from those with cystic fibrosis) develop diabetes mellitus [38].

#### 2.4.2. Proteins

With reduced glycogen stores in CLD, proteins are increasingly utilized for gluconeogenesis [39]. However, the liver’s capacity for protein synthesis in CLD is limited due to reduced substrate availability, decreased hepatocyte function, and increased catabolism. Consequently, hypoalbuminemia can develop, leading to peripheral edema, ascites, and decreased enteral intake due to discomfort [13]. Additionally, the liver is the site of synthesis for all coagulation factors with the exception of factor VIII, thus coagulopathy is also a consequence of CLD [30]. Increased protein catabolism also results in increased nitrogenous waste products such as ammonia, which is normally converted to urea by the liver for excretion. This conversion is compromised in CLD, resulting in increased ammonia levels. Abnormal protein use by the liver in CLD results in increased aromatic amino acids (AAAs) and decreased branched-chain amino acids (BCAAs) [40]. An abnormal ratio of BCAAs to AAAs correlates with both histologic damage and encephalopathy [40]. Increased cerebral uptake of AAAs results in formation of false neurotransmitters and causes neurologic dysfunction, along with increased ammonia levels [41]. That said, children with CLD usually do not require protein restriction due to their increased growth needs [42].

#### 2.4.3. Fats

Cholestasis in CLD results in compromised absorption of fats due to decreased delivery of bile salts to the small bowel [43]. This can further be aggravated by small bowel bacterial overgrowth, such as in children who have undergone a Kasai portoenterostomy for biliary atresia, as the bacteria unconjugate bile salts [44]. Congested gastric and intestinal mucosa from portal hypertension further worsen fat malabsorption, and medications, such as cholestyramine to treat pruritus, bind bile salts, decreasing micellar solubilization and thus absorption of di- and monoglycerides from long-chain triglycerides (LCTs) [30]. Children with Alagille syndrome can also have pancreatic insufficiency and decreased lipase, which compromises the hydrolysis of triglycerides [45]. Due to these mechanisms, up to 50% of LCTs, fat-soluble vitamins, and essential polyunsaturated fatty acids (PUFAs) may not be absorbed well in children with CLD [46,47,48] and deficiencies in long-chain polyunsaturated fatty acids (LCPs) critical to neurologic growth and development, such as arachidonic acid and docosahexaenoic acid (DHA), can develop within 8–12 weeks [30]. Furthermore, children with CLD have increased fat oxidation, likely due to decreased carbohydrate stores. This increased oxidation further decreases fat stores, which then are difficult to replete given the presence of fat malabsorption [13,29]. Lastly, impaired bile flow from cholestasis can result in increased plasma concentration of triglycerides and cholesterol, which may then deposit in the hands, elbows, knees, ankles, and corneas as xanthomas [49].

### 2.5. Fat-Soluble Vitamins

CLD affects absorption, metabolism, and storage of fat-soluble vitamins. Decreased delivery of bile salts to the small bowel results in malabsorption of fat, as well as the fat-soluble vitamins A, D, E, and K. Without supplementation, fat-soluble vitamin deficiency can develop within 6–12 weeks of birth [30] and even after supplementation up to 30% of severely cholestatic children will remain deficient in all fat-soluble vitamins [50,51].

#### 2.5.1. Vitamin A

Vitamin A has the activity of all-*trans* retinol. Retinol is required for rhodopsin formation (a pigment required for retina rod cell function and dark adaptation), as well as normal cell differentiation. In the diet, vitamin A is available as retinyl palmitate from animal sources (dairy, eggs, fish oils) and as carotenoids from plants (leafy green vegetables, orange-colored fruits, vegetables). Decreased bile salts in the intestine results in decreased hydrolysis of retinyl esters to retinol, as well as decreased formation of micelles, which are required for absorption. Retinol-binding protein (RBP), which is synthesized by the liver and transports vitamin A to peripheral tissues, is also decreased in CLD, thus impairing utilization. Vitamin A deficiency can result in night blindness, xerophthalmia, and keratomalacia [13].

#### 2.5.2. Vitamin D

Vitamin D consists of a group of fat-soluble prohormones and their metabolites, the chief of which are vitamin D_2_ (ergocalciferol) and vitamin D_3_ (cholecalciferol). These must first undergo hydroxylation in the liver and kidney before they can be utilized by the body. Vitamin D helps regulate calcium and phosphorus, and is critical in bone homeostasis. It can be synthesized in the skin when exposed to sunlight, and can be consumed in the diet from fish oils and fortified dairy products. In CLD, vitamin D deficiency develops from malabsorption, decreased dietary intake and sunlight exposure, and compromised liver hydroxylation. Deficiency results in defective bone mineralization, and if untreated, rickets and fractures [13]. Infants are particularly susceptible, as their bone mineral content can decrease rapidly over the first two years of life [52]. Breastfed infants with CLD are at even higher risk, as breastmilk contains low amounts of vitamin D [53].

#### 2.5.3. Vitamin E

Vitamin E, which consists of tocopherols and tocotrienols, has important anti-oxidant properties. Vitamin E is found in leafy green vegetables, vegetable oils, and nuts. Deficiency in vitamin E in CLD results from malabsorption, and consequences thereof include problems with nerve conduction, including peripheral neuropathy, myopathy, and spinocerebellar dysfunction. Vitamin E deficiency can also result in hemolytic anemia due to oxidative damage to the membranes of red blood cells [54].

#### 2.5.4. Vitamin K

Vitamin K is necessary for carboxylation of glutamic residues on coagulation factors II, VII, IX, and X, as well as proteins C and S, within the liver. Vitamin K_1_ [phylloquinone] is found in leafy green vegetables and dairy products, and vitamin K_2_ [menaquinone] is synthesized by intestinal bacterial flora. Deficiency in vitamin K in CLD results from malabsorption and manifests as coagulopathy, with easy bleeding and bruising [13]. Due to limited ability of the body to store vitamin K, vitamin K deficiency is one of the earliest fat-soluble vitamin deficiencies to develop in children with CLD [55].

### 2.6. Trace Elements and Metals

Derangements in trace elements and metals can occur with CLD. Calcium and magnesium are often depleted in CLD, as vitamin D stimulates their absorption from the intestines. They also bind to unabsorbed fatty acids, which further decreases their absorption in children with CLD. Iron deficiency can occur from recurrent gastrointestinal bleeds [13]. Iron deficiency can result in compromised neurologic development in children [56] and multiple studies have found that children with CLD demonstrate delays in mental (−1 standard deviation) and motor (−2 standard deviations) function [6]. Zinc deficiency can develop due to malabsorption as well as increased urinary losses, as zinc is retained in the body by being bound to albumin [57]. Deficiency in zinc results in acrodermatitis, immunodeficiency, and altered protein metabolism; in addition, zinc deficiency, as well as selenium deficiency, can exacerbate growth failure and poor protein synthesis [22,58]. In contrast, copper and manganese levels may be increased in children with cholestasis and CLD, as they are excreted in the bile [59,60]. Thus, in children with CLD who are receiving total parenteral nutrition (TPN), the amount of manganese they receive should be closely monitored, as manganese toxicity in the form of deposition in the basal ganglia can develop [61,62].

## 3. Strategies to Manage Malnutrition in Cld

### 3.1. Nutritional Assessment

Accurate nutritional assessment is critical to the management of children with CLD. Standard weight and height measurements may be inaccurate in children with CLD, as they can be confounded by fluid overload, ascites, and organomegaly [30]. Body weight alone may underestimate the incidence of malnutrition in adults and children with CLD by up to 50% [63]. Linear growth is a more sensitive parameter, but stunting and growth deceleration do not occur until late in growth failure. Thus, other measurements, such as triceps or subscapular skinfolds, midarm circumference, and arm muscle measurements (midarm muscle area), should be used. Triceps, skinfolds, and midarm circumference are indicators of body fat and protein, and can reveal early loss in fat stores before height and weight are affected. In children with CLD, triceps skinfold thickness has been shown to be more sensitive for malnutrition compared to weight-for-height *z* scores [14,16]. Furthermore, these upper limb measurements are less affected by edema compared to truncal or lower limb measurements [13]. When growth data are documented, they should be charted as standard deviation scores related to the median value for the child’s age and sex, where a *z*-score of 0 is equivalent to the 50th percentile. This will assist in evaluating whether nutritional interventions are effective. Children who are particularly at risk for developing malnutrition include those younger than two years in age with severe cholestasis (bilirubin > 4 mg/dL), progressive liver diseases (biliary atresia and severe familial intrahepatic cholestasis), end-stage liver disease awaiting liver transplantation, and recurrent complications of liver disease (ascites, bleeding varices) [30].

Protein markers, such as albumin and prealbumin, are of limited utility in children with CLD. Albumin may be depressed due to hepatic synthetic dysfunction, inflammation, or acute physiologic stress [64]. Prealbumin is a more sensitive marker of malnutrition compared to albumin, as it has a shorter half-life (2 days compared to 18–20 days); that said, prealbumin levels can be normal in chronic malnutrition [65]. On the other hand, essential fatty acid deficiency can be diagnosed with an increased serum triene to tetraene ratio (greater than 0.4). Essential fatty acid deficiency may exist in up to a third of children with end-stage liver disease awaiting liver transplant [22]. Although there are drawbacks to each of the individual assessment tools, a combination of the tools described can be used to more clearly evaluate malnutrition. 

### 3.2. Supplementation of Specific Macronutrients and Micronutrients

Because of the increased energy needs of children with CLD, energy intake should be increased to 140–200% of estimated average requirements (EARs). In infants, this can be achieved by concentrating medium-chain triglyceride (MCT)-containing formulas (an example being Pregestimil by Mead Johnson Nutrition), so as to increase the number of kilocalories per ounce. Older children can be supplemented with high-calorie, nutrient-dense drinks (examples including Pediasure Peptide by Abbott Nutrition, Peptamen by Nestle Healthcare Nutrition). If a sufficient amount cannot be consumed orally, nasogastric (NG) feedings may be needed [30]. Despite disruption to the GH/IGF-I axis, GH therapy has not been found to have benefits in children with end-stage liver disease [66,67].

#### 3.2.1. Carbohydrates

Carbohydrates are a major source of energy, and can be particularly useful for increasing caloric intake. They can be given as monomers, polymers, and starch. Complex carbohydrates such as maltodextrin and glucose polymers can be particularly useful, as their use restricts the osmolality of feeds, while maintaining a high-energy density greater than 1 kcal/mL. In infants, glucose polymers can be added to feeds, while in older children, a supplemental drink can be given, or mixed with fluids and foods [30].

#### 3.2.2. Proteins

Protein restriction is rarely needed in children or adults with CLD [68]. Children with CLD require 2–3 g/kg/day of protein, but can tolerate up to 4 g/kg/day without developing encephalopathy [42]. Severe protein restriction (<2 g/kg/day) may be temporarily required in the context of acute encephalopathy, but should not be continued long-term, as this can lead to endogenous muscle protein consumption [27]. 

Given the abnormal AAA to BCAA ratio in children with CLD, there has been interest in whether BCAA-enriched formulas can have nutritional benefit. Specific hypercaloric formulas with low salt and lactose, high MCT and BCAA are available [27]. Thus far there is insufficient evidence to recommend routine use of BCAA-enriched formulas, though there have been studies showing potential benefit. One study which compared children receiving 32% BCAA formula compared to standard formula showed improved lean body mass, though no improvement in amino acid levels [69]. Another study evaluated infants receiving 50% BCAA formula compared to 22% BCAA formula, and demonstrated that infants receiving the 50% BCAA formula had improved protein retention due to suppressed endogenous protein catabolism and normalization of the plasma amino acid profile [28]. Animal models of biliary obstruction have increased weight gain, protein and muscle mass, body composition, and bone mineral density if given BCAAs [70]. That said, numerous studies in adults have not demonstrated a clear benefit to BCAA supplementation [71].

#### 3.2.3. Fats

MCTs, unlike LCTs, do not require micellar solubilization to be transported into the enterocyte, and are transferred directly into the enterocyte and to the portal circulation without reesterification [72]. 95% of MCTs are absorbed even in very cholestatic children, thus MCTs are critical in managing nutrition in children with CLD, where absorption of LCTs is highly compromised [73]. Although 30–50% of total fat should be provided as MCTs [13], care should be taken to ensure LCTs are not eliminated from the diet, as they provide essential fatty acids. Thus, it is necessary to increase overall total fat intake, in both LCTs and MCTs. For older children, MCT oil and emulsions can be added to meals, and should be balanced by fats high in LCP content [30]. For infants, formulas containing up to 75% fat as MCT can be given, but formulas with >80% MCTs can lead to essential fatty acid deficiency. Increased MCT content can also worsen steatorrhea [74]. The minimal linoleic acid intake recommended for infants is 1–2% of total energy intake, with a ratio of linoleic to linolenic acid of 5:15.1 [75]. They can be supplemented in the form of walnut or fish oils, as well as dietary products rich in PUFAs, such as egg yolks [30].

### 3.3. Fat-Soluble Vitamins

In the presence of direct-reacting serum bilirubin levels greater than 2 mg/dL, the diet should be adequately supplemented with fat-soluble vitamins [76]. The role of serum bile acid as a surrogate marker to guide the monitoring of fat-soluble vitamin deficiency is still undefined. In infants with biliary atresia, total serum bilirubin appears to be a better predictor of fat-soluble vitamin deficiency compared to serum bile acids, though neither are perfect [77]. Serum vitamin and prothrombin levels should be monitored to allow proper adjustment of dosages to the specific needs of the patient [78].

#### 3.3.1. Vitamin A

**Figure 2:**
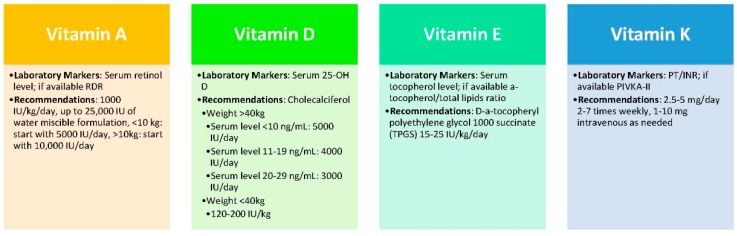
Management of fat-soluble vitamin deficiency in CLD. INR: international normalized ratio; IU: international unit; PIVKA-II: protein induced in vitamin K absence; PT: prothrombin time; RDR: retinol dose response.

Serum retinol level is the most convenient and practical means of measuring vitamin A status, though retinol dose response (RDR) is believed to be more reliable but is not widely available (Figure 2). It is important to monitor levels in children receiving supplementation, as hypervitaminosis A can lead to potentially fatal hepatotoxicity [13]. Supplementation with 5000–10,000 IU/day may be needed in children with CLD [30].

#### 3.3.2. Vitamin D

Serum 25-OH D is the most abundant vitamin D metabolite in the body, and can be used to monitor vitamin D status. Low levels of 25-OH D are associated with reduced bone mineral density in children with CLD [52]. It is important to optimize vitamin D levels before liver transplantation due to the use of corticosteroids post-transplant, which can compromise bone density [79]. Co-supplementation with a micellar vitamin E formulation can improve vitamin D absorption in children with cholestatic liver disease [80] and 25-OH D_3_ is more water-soluble and thus better absorbed compared to vitamin D_2_ [81]. 25-OH D levels, along with calcium and phosphorus, should be monitored to prevent vitamin D overdose. Up to 400 IU/day can be given as supplementation; however, children who are deficient may need substantially higher doses [13].

#### 3.3.3. Vitamin E

The serum tocopherol level is commonly used to measure vitamin E status, though the alpha tocopherol/total lipids ratio is more specific [82]. d-alpha-tocopheryl polyethylene glycol 1000 succinate (TPGS) is the most readily absorbed form of vitamin E in cholestatic patients, as it can form micelles without the need for bile salts [13]. Unfortunately, correction of vitamin E deficiency may not reverse severe spinocerebellar degeneration [83], but it can reverse most other neurologic complications [84]. Supplementation with 50–400 IU/day of TPGS may be needed in children with CLD [30].

#### 3.3.4. Vitamin K

The PIVKA-II [protein induced in vitamin K absence] assay is the most sensitive for measuring vitamin K deficiency, but it is not widely available [50,85]. Therefore, vitamin K status is usually evaluated by checking coagulation values, including prothrombin time (PT) and international normalized ratio (INR). Deficiency can be diagnosed if these values improve after a dose of parenteral vitamin K [13]. Supplementation with 2.5–5 mg/day of vitamin K may be needed [30], though oral vitamin K, even in micellar form, is poorly absorbed in children with CLD [86]. This is further exacerbated by medications commonly used to treat encephalopathy, such as lactulose, which reduces intestinal bacterial production of vitamin K. Thus, intermittent parenteral repletion may be required [13]. 

### 3.4. Water-Soluble Vitamins and Minerals

Water-soluble vitamins should also be supplemented in children with CLD in the form of a multivitamin. For minerals, including selenium, zinc, calcium, and magnesium, supplementation should be based from plasma levels. Iron may need to be supplemented in children with chronic blood loss from gastrointestinal bleeds [30]. Repletion of zinc and magnesium in particular may be helpful, as zinc plays a role in immune function and tissue repair [13], while magnesium may help improve bone status [87].

### 3.5. Mode of Delivery

Nutritional supplementation should be given enterally whenever possible. Enteral nutrition has numerous advantages over parenteral nutrition: it is cheaper, more physiologic, does not come with the risk of catheter-associated bloodstream infections, maintains gastrointestinal tract immunity as well as gut barrier integrity, and reduces bacterial overgrowth. However, as discussed in a previous section, many children with CLD are unable to orally consume sufficient calories to treat or prevent malnutrition. Therefore, NG tube feedings are often required. Gastrostomy tubes are generally avoided in children with CLD due to portal hypertension and the potential for stomal varices to develop, placement difficulty due to organomegaly, and risk of peritoneal infection with ascites [8,68]; they may be helpful in children with stable compensated liver disease such as cystic fibrosis [30]. With modern soft Silastic™ (Dow Corning Corporation, Midland, MI, USA) NG tubes, NG feeds are safe even in patients with esophageal varices [42,88,89,90]. Using a NG tube for nocturnal feeds is usually the first choice, as it allows for normal oral feeding during the day while supplementing overnight. Nocturnal feeding is also helpful in infants with severe CLD, as it prevents fasting hypoglycemia and reduces protein catabolism. Children with severe malabsorption or feeding intolerance may require continuous feeds [30]. Intensive enteral NG feeding can successfully reverse malnutrition in children with CLD, as well as decrease parental anxiety [42,73,91]. However, feeding aversion can also develop, especially in infants who have received long-term NG feeds. This is further aggravated by the need for often unpalatable medication and formulas. Thus, involvement of a multidisciplinary care team including dieticians, psychologists, and speech and occupational therapists is critical. Strategies to prevent feeding aversion include promoting daytime oral intake, as well as encouraging children to experiment with various age-appropriate flavors and textures [73].

Some children with CLD will require parenteral nutrition. These include children who cannot tolerate enteral nutrition due to feeding intolerance, or those with recurrent variceal bleeding. In the short-term, parenteral nutrition is not associated with hepatobiliary dysfunction or worsened cholestasis, though these certainly do occur with long-term use [92]. Standard amino acid and lipid formulations are well tolerated in patients with stable CLD, though triglyceride levels should be closely monitored in children with severe liver disease, hepatic encephalopathy, and sepsis. Amino acid levels should also be monitored. If encephalopathy does develop, the amino acid content can be decreased to 1–2 g/kg/day [30]. Manganese levels also need to be monitored given the potential for manganese toxicity to exacerbate CLD [93,94]. Parenteral nutrition is particularly helpful in children with acute fulminant liver failure, as these children are in a hypercatabolic state. In these children, standard formulations can be used, but the total volume should be restricted to 75% of maintenance, and concentration may need to be further increased to prevent hypoglycemia. Protein restriction should not be needed, especially if the patient is being electively ventilated [30].

## 4. Effect of Liver Transplantation on Nutritional Status [Figure 3]

**Figure 3:**
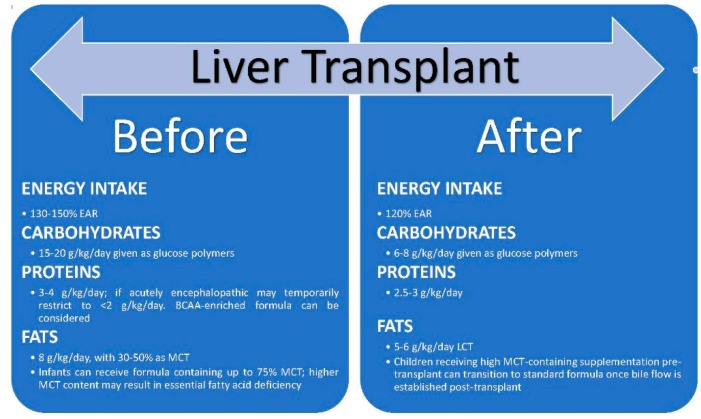
Nutritional needs of children with CLD before and after liver transplant. EAR: estimated average requirements; LCT: long-chain triglycerides; MCT: medium-chain triglycerides.

Malnutrition is a significant risk factor for both morbidity and mortality related to liver transplantation, thus nutritional support is of utmost importance in children with CLD prior to undergoing transplant (Figure 3) [15,95,96]. Children who were malnourished prior to transplant may require support with parenteral nutrition peri-operatively, while children with normal nutritional status prior to transplant can start enteral feedings with rapid buildup of calories within 3–5 days postoperatively. Children after liver transplant require at least 120% of EAR post-operatively, which can be administered in the form of high-energy pediatric and infant formulas, either orally or via nasogastric tube [30]. Children with oral aversion pre-operatively will likely require nasogastric supplementation post-operatively for up to two months; regular diet for age is usually achieved by six months [97]. Energy intake should include 6–8 g/kg/day of carbohydrates, 2.5–3 g/kg/day of protein, and 5–6 g/kg/day of fat [30].

Preoperative nutritional status also affects postoperative growth. Children who are more stunted, with a height standard deviation score (SDS) >−2, grow more rapidly after a transplant, but may not achieve normal height. This is in contrast to children who are less stunted: while their initial growth velocity post-operatively is slower, they ultimately achieve normal growth velocity [98]. There is no difference in catch-up growth between genders or pre-transplantation liver disease; the exception being Alagille syndrome, which is associated with growth failure, both before and after a transplant, in up to 50% of patients [99,100]. Recombinant GH has been trialed in eight growth-retarded children after a transplant, and while there were increases in both median growth rate and height SDS, growth velocity was not maintained beyond the first year [101].

Ultimately, nutritional rehabilitation can be achieved with liver transplantation [73,102,103]. After transplantation, there is a rapid return to normal midarm muscle area and midarm fat within 3–6 months [103,104] and catch-up growth is usually achieved within 18 months [15]. However, it can take months to years for bone density to return to normal. Osteoporosis and fractures have been described within 3–6 months after a transplant, and can be exacerbated by glucocorticoid therapy required after a transplant [67]. Long-chain PUFA and BCAA metabolism also take time to normalize after a transplant [71,105] and abnormalities in IGF-binding proteins BP1, BP2, and BP3 persist for several months [106]. 

## 5. Conclusions

Malnutrition is common in children with CLD, and requires aggressive and appropriate management to correct nutritional deficiencies. Infants with CLD are particularly susceptible to malnutrition given their low reserves. Children with CLD would benefit from early intervention by a multi-disciplinary team, to try to achieve nutritional rehabilitation, as well as to optimize outcomes for liver transplantation.
